# Psychological Distress and Post-Traumatic Symptomatology among Dental Healthcare Workers in Russia: Results of a Pilot Study

**DOI:** 10.3390/ijerph18020708

**Published:** 2021-01-15

**Authors:** Maria Sarapultseva, Alena Zolotareva, Igor Kritsky, Natal’ya Nasretdinova, Alexey Sarapultsev

**Affiliations:** 1 Department of Pediatric Dentistry, Medical Firm Vital EBB, 620144 Ekaterinburg, Russia; m.sarapultseva@gmail.com; 2 Ural Division of Russian Academy of Sciences, Institute of Immunology and Physiology (IIP), 620002 Ekaterinburg, Russia; igor81218@gmail.com; 3 International Laboratory of Positive Psychology of Personality and Motivation, Department of Psychology, National Research University Higher School of Economics, 101000 Moscow, Russia; 4 Institute of Natural Sciences and Mathematics, Ural Federal University Named after the First President of Russia, 620026 Ekaterinburg, Russia; 5 Autonomous Non-Commercial Organization «Association Stomatology», 620102 Ekaterinburg, Russia; nataweb@mail.ru; 6 School of Medical Biology, South Ural State University, 454080 Chelyabinsk, Russia

**Keywords:** anxiety, COVID-19, DASS-21, dentistry, depression, IES-R, infection, PSS-SR, PTSD, Russia

## Abstract

The spread of SARS-CoV-2 infection has increased the risk of mental health problems, including post-traumatic stress disorders (PTSD), and healthcare workers (HCWs) are at greater risk than other occupational groups. This observational cross-sectional study aimed to explore the symptoms of depression, anxiety, and PTSD among dental HCWs in Russia during the coronavirus disease 2019 (COVID-19) pandemic. The survey was carried out among 128 dental HCWs from three dental clinics of Ekaterinburg, Russia. The mean age of the sample was 38.6 years. Depression, anxiety, and stress were assessed using the Depression Anxiety and Stress Scale-21 (DASS-21); PTSD was assessed using the PTSD Symptom Scale-Self-Report (PSS-SR); subjective distress was assessed using the Impact of Event Scale-Revised (IES-R). The results indicated that 20.3–24.2% HCWs had mild to extremely severe symptoms of psychological distress, and 7.1–29.7% had clinical symptoms of PTSD. No differences between females and males were revealed. HCWs working directly with patients had significantly higher levels of PTSD symptoms and the risk of PTSD development compared to those working indirectly, whereas older HCWs had significantly higher levels of both psychological distress and PTSD symptoms compared to younger HCWs. Thus, dental HCWs are at high risk for psychological distress and PTSD symptoms during the COVID-19 pandemic.

## 1. Introduction

The global spread of SARS-CoV-2 infection has deeply affected the world. The increasing numbers of patients and outbreak-affected countries have elicited public worry and thus increased the risk of mental health problems (insomnia, anxiety, depression, and stress-related disorders, including the post-traumatic stress disorder (PTSD)) [1,2,3,4,5,6].

The psychological burden of healthcare workers (HCWs) has received heightened awareness, with research continuing to show high rates of mental disorders among them in most countries, including China, the U.K., U.S.A., India, and Italy [3,4,5,6]. Thus, nearly 58% of HCWs in the U.K. met the threshold for clinically significant PTSD, anxiety, or depression [3]; in China, the prevalence rates of these conditions were estimated at 9.8–50.4%, 27.1–44.6%, and 15.0–25.0%, respectively [7,8]; and in Italy, about 22% and 40% of HCWs reported moderate-to-severe symptoms of anxiety and PTSD, respectively [5]. In the U.S.A. and Australia, more than half of HCWs screened positive for PTSD symptoms, almost half screened positive for depression, and one-third screened positive for anxiety [9,10]. Overall, the substantial proportion of HCWs self-reported moderate-to-severe symptoms of depression (13.5–44.7%;), anxiety (12.3–35.6%), and PTSD (7.4–37.4%) [6,9].

Dentists may have a higher risk for SARS-CoV-2 transmission than the average HCWs. This is because dental healthcare delivery requires close physical contact between patients and specialists, while dental procedures generate aerosols, which pose potential risks to operators and patients [11]. Moreover, for these reasons, severe restrictions in dental practice have been adopted by governments to avoid this source of contagion [12,13]. In several countries, dentists have been allowed to practice only emergency/urgent procedures during the whole period of the lockdowns [12,14,15]. The obtained perceived job insecurity has additionally affected their mental health [16,17,18], having been positively associated with depressive symptoms [12]. This is exemplified by the results of cross-sectional surveys among dentists from China, India, Israel, Italy, and the U.K., which indicated elevated levels of subjective overload, psychological distress, and anxiety among participants [19,20]. Thus, more than two-thirds of the general dental practitioners (78%) from 30 countries questioned in the online survey of Ammar et al. (2020) were anxious and scared by the devastating effects of coronavirus disease 2019 (COVID-19) [20]. During the lockdown, about half of Indian dentists exhibited a higher degree of perceived stress in comparison to the general population [21]. The elevated levels of depression, anxiety, and stress were recorded at 60.64%, 37.02%, and 34.92%, respectively, among the dental students of Saudi Arabia [22]. Generally, about 9–11.5% of dentists were heavily affected by the pandemic and reported severe anxiety and stress [20,23,24].

Thus, despite having a high standard of knowledge and practice, dental practitioners around the globe are in a state of anxiety and fear while working during the COVID-19 pandemic [25]. Given the above, this study aimed to explore the symptoms of depression, anxiety, and PTSD among dental HCWs in Russia during the COVID-19 pandemic.

## 2. Materials and Methods 

### 2.1. Study Design

Our study had a cross-sectional design and was carried out between September 1 and September 20, 2020, among dental HCWs from Ekaterinburg, Russian Federation. The total number of dentists in the Russian Federation is about 63,700 (approximately 4.3 per 10,000 population), and the number of dental technicians is 15,300 (approximately 1 per 10,000 population) [26]. The total number of dentists in Ekaterinburg is about 1110, while the estimated number of dental technicians is about 380. 

To be eligible for participation, respondents had to meet the following inclusion and exclusion criteria. The inclusion criteria included: (1) working in a dental clinic during the COVID-19 pandemic, defined as the period from March 25, 2020; (2) professional activity from before the coronavirus epidemic, i.e., before January 2020; (3) providing informed consent to participate in the study through the response “Yes”. The exclusion criteria included: (1) being on sick leave, maternity, parental or care to leave before the announcement of the epidemic in Russia; and (2) withdrawal from work for health reasons. There was no target recruitment size. Direct comparisons were not drawn; therefore, a power calculation was not performed. 

**Figure 1:**
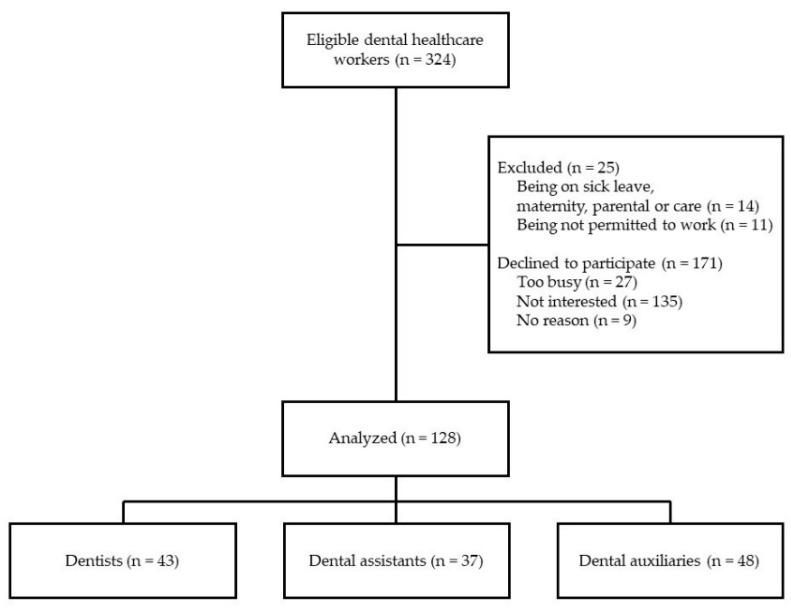
Flow diagram for study participants.

During the study, 324 HCWs from three dental clinics met eligibility criteria, and 128 completed the study. The participants were divided into three groups: a “Dentists” group, consisting of 43 HCWs with an MD degree; a “Dental assistant” group, consisting of 37 dental HCWs without an MD degree; and a “Dental auxiliary” group, consisting of 48 HCWs, including dental laboratory technicians, front desk receptionists, and nurse aides (Figure 1). The HCWs in the first two groups were considered as “face-to patient HCWs”, while the “Dental auxiliary” group consolidated HCWs who did not have direct contact with patients. The latter division was conducted because direct patient care has been shown to be among the factors related to poor mental health outcomes in HCWs during infectious disease outbreaks [27,28,29].

### 2.2. Ethics Approval

Ethical approval to the project (meeting # 6 of the Ethics Commission of the Academic Council of the University) was obtained from the Chelyabinsk State University (Chelyabinsk, Russia).

### 2.3. Measures

All participants completed the sociodemographic form and the set of measures. The set of self-report measures used in this study was determined by analogy with previous studies aimed to assess the mental health of HCWs during an epidemic [30]. The set included the DASS-21 measure [31,32,33], and the complex of the IES-R [7,32,34], with the PSS-SR measures as PTSD screening tools [35].

The Depression Anxiety and Stress Scale-21 (DASS-21) is a 21-item self-report measure assessing three dimensions: depression (i.e., anhedonia, dysphoria, hopelessness, devaluation of life, self-deprecation, lack of interest or involvement, and inertia), anxiety (i.e., autonomic arousal, skeletal muscle effects, situational anxiety, and subjective experience of anxious affects), and stress (i.e., difficulty in relaxing, nervous excitation, states of being easily upset/agitated, irritable/over-reactive and impatient) [36]. A 4-point severity scale measures the extent to which each state has been experienced over the past week via rating each item on a four-point Likert scale from 0 (“never applied to oneself”) to 3 (“very much or most of the time”). On the DASS-21 depression subscales, scores of 0–4 were deemed as “normal”, 10–13 as “mild”, 14–20 as “moderate”, 21–27 as “severe”, and 28–42 as “extremely severe” depression. The DASS-21 anxiety subscale scores were assessed as “normal” (0–3), “mild” (4–5), “moderate” (6–7), “severe” (8–9), and extremely severe” (10–21). The DASS-21 stress subscale scores were divided into “normal” (0–7), “mild” (8–9), “moderate” (10–12), “severe” (13–16), and “extremely severe” (17–21) stress [37]. The version of DASS-21 previously utilized to examine a Russian community sample was used in the study [38]. The Cronbach’s alpha coefficients in the present study were 0.79, 0.76, 0.87 for depression, anxiety, and stress, respectively, and 0.92 for the total score. 

The Impact of Event Scale-Revised (IES-R) is a 22-item measure assessing subjective distress caused by traumatic events. The measure contains three subscales: intrusion (i.e., intrusive thoughts, nightmares, intrusive feelings, and imagery), avoidance (i.e., numbing of responsiveness, avoidance of feelings, situations, and ideas), and hyperarousal (i.e., anger, irritability, hypervigilance, heightened startle) [39]. Respondents rate the frequency of symptoms on a scale from 0 (“not at all”) to 4 (“extremely”). The total IES-R score was graded for severity from normal (0–23), mild (24–32), moderate (33–36), and severe psychological impact (37–88) [40]. The Russian version of the IES-R examined in both clinical and non-clinical samples was used in the study [41]. The Cronbach’s alpha coefficients in the present study were 0.87, 0.84, 0.78 for intrusion, avoidance, and hyperarousal, respectively, and 0.93. for the total score.

The PTSD Symptom Scale-Self-Report (PSS-SR) is a 17-item measure assessing the presence and severity of post-traumatic stress disorder (PTSD) symptoms according to DSM-IV criteria [42]. The measure contains three subscales: re-experiencing (i.e., recurrent and intrusive distressing recollections of the event), avoidance (i.e., efforts to avoid thoughts, feelings, or conversations associated with the event), and increased arousal (i.e., difficulty falling or staying asleep) [29]. Respondents rate the frequency of PTSD symptoms on a scale from 0 (“not at all”) to 3 (“3 to 5 or more times per week/very much/almost always”). The total PSS-SR score is used to identify normal (0–9), moderate (10–19), and severe (20–51) PTSD symptoms. A cut-off score of 14 was used for the total PSS-SR [43]. The PSS-SR was translated into Russian using forward-translation and back-translation procedures. The Cronbach’s alpha coefficients were 0.79, 0.78, 0.72 for re-experiencing, avoidance, and increased arousal, respectively, and 0.93 for the total score in the current study.

### 2.4. Statistical and Data Analysis

The prevalence rates of psychological distress and PTSD symptoms were derived according to the cut-off values for the total DASS-21, PSS-SR, and IES-R scores. Descriptive statistics were calculated for categories and sociodemographic characteristics. The distribution of DAS-21, PS-SR, and IES-R scores among the groups was abnormal (Shapiro–Wilk normality test >0.05); therefore, nonparametric statistical methods (Mann–Whitney U and Kruskal–Wallis H tests) were used.

Finally, multiple regression analysis was performed to study the impact of socio-demographic characteristics and psychological distress on the severity of PTSD symptoms. All statistical analyses were performed using the software IBM SPSS version 27 (IBM Corporation, Armonk, NY, USA) and Microsoft Excel version 14.0 (Microsoft Corporation, Redmond, WA, USA).

## 3. Results

### 3.1. Participant Demographic Characteristics

In the present study, 128 HCWs were included. Of the participants, 43 (33.6%) were dentists, 37 (28.9%) were dental assistants, and 48 (37.5%) were other healthcare workers (dental laboratory technicians, front desk receptionists, and nurse aides). Most of the participants were female (101 (78.9%)), and 80 (62.5%) had direct contact with patients. The mean age of the sample was 38.6 years (SD = 13.9). The participant demographic characteristics are presented in Table 1.

### 3.2. Prevalence of Psychological Distress and PTSD in Healthcare Workers

Table 2 presents the prevalence of psychological distress and PTSD symptoms in HCWs. In the sample, 20.3% had mild to extremely severe symptoms of depression, 24.2% had mild to extremely severe symptoms of anxiety, and 24.2% had mild to extremely severe symptoms of stress, determined using established cut-off scores for the DASS-21. Furthermore, 29.7% had moderate or severe PTSD symptoms using established cut-off scores for the PSS-SR, and 7.1% had mild to severe PTSD symptoms using established cut-off scores for the IES-R.

The descriptive statistics for DASS-21, PSS-SR, and IES-R scores are presented in Table 3.

The Mann–Whitney U test showed no differences between females and males on the DASS-21, PSS-SR, and IES-R scores. 

The Kruskal–Wallis H test showed that HCWs aged 51–64 years, in comparison to HCWs aged 18–35 years and 36–50 years, had significantly higher DASS-21 depression scores (H = 10.47, *p* < 0.01), DASS-21 anxiety scores (H = 9.37, *p* < 0.01), DASS-21 stress scores (H = 7.83, *p* < 0.05), DASS-21 total scores (H = 10.54, *p* < 0.01), PSS-SR increased arousal scores (H = 6.70, *p* < 0.05), IES-R avoidance scores (H = 7.72, *p* < 0.05), and IES-R total scores (H = 7.21, *p* < 0.05). 

The Mann–Whitney U test showed that HCWs who had direct contact with patients, in comparison with those who had no contacts, had significantly higher PSS-SR re-experiencing scores (U = 1503.0, z = −2.14, *p* < 0.05), PSS-SR total scores (U = 1482.5, z = −2.16, *p* < 0.05), IES-R intrusion scores (U = 1462.5, z = −2.35, *p* < 0.05), IES-R avoidance scores (U = 1491.5, z = −2.19, *p* < 0.05), and IES-R total scores (U = 1512.0, z = −0.2.03, *p* < 0.05). 

Finally, the Kruskal–Wallis H test showed no differences between dentists, dental assistant, and dental auxiliaries, but pairwise comparisons using a Mann–Whitney U test showed that dentists, in contrast to other HCWs, had significantly higher PSS-SR re-experiencing scores (U = 762.5, z = −2.24, *p* < 0.05), PSS-SR increased arousal scores (U = 763.5, z = −0.2.16, *p* < 0.05), PSS-SR total scores (U = 733.0, z = −2.38, *p* < 0.05), and IES-R intrusions scores (U = 776.0, z = −2.15, *p* < 0.05). Furthermore, dental assistants, in contrast to other HCWs, had significantly higher IES-R avoidance scores (U = 663.0, z = −2.09, *p* < 0.05).

### 3.3. Risk Factors for PTSD Symptoms Development

Multiple regression analysis was used to examine the potentially varying influence of psychological distress and sociodemographic characteristics on the severity of PTSD symptoms (Table 4). Sex, working position, age, depression, anxiety, and stress were used as predictors, and PTSD symptoms were used as the predicted variables (PSS-SR and IES-SR). The predictor “occupation” was excluded from the linear regression model because it was collinear with the “working position”. Model 1 was statistically significant, predicting 60% of the variance in PSS-SR scores (F_[7, 120]_ = 25.93, multiple R = 0.60, adjusted R^2^ = 0.58). Model 2 was also statistically significant, predicting 48% of the variance in IES-R scores (F_[7, 120]_ = 16.02, multiple R = 0.48, adjusted R^2^ = 0.46). In both models, the predictors “stress” and “working position” had a significant effect on PTSD symptoms.

## 4. Discussion

Emerging evidence from research on the COVID-19 pandemic indicates high rates of mental disorders among HSCWs in most countries, including China, the U.S.A., India, and Italy [2,3,16,18,23,24,44,45].

According to the obtained results in this study, 21.9% of the sample had moderate to severe symptoms of psychological distress using an established cut-off for the DASS-21, which was similar to the results (23.6%) of an online survey among the HCWs of various specialties of Russia [36].

The revealed stress levels were significantly higher than those described among dental academics (9.9% using an established cut-off for the IES) [20] and Israeli dentists (11.5% using Kessler’s K6 Distress Scale) [24]. With that, the distress levels were significantly lower than in India, where about 50% of dentists had distress (using an established cut-off for the COVID-19 Peritraumatic Distress Index, CPDI) and 80% had perceived stress, as indicated by the PSS [21]; and Saudi Arabia, where 34.92% of dental students had elevated levels of stress (using an established cut-off for the DASS-21 [22]. This discrepancy could be attributed to the use of various scales (such as CAD-7, CPDI, DASS-21, and IES-R) for measuring distress and anxiety levels, to a period of the pandemic, when the studies were conducted, and even with the differences in the dental care systems and government restrictions in various countries. Thus, a strong dose–response relationship was observed in the association between country-level fatality rate and stress levels; a higher fatality rate was associated with higher odds of severe and moderate stress [20]. The emotional and mental characteristics of HCWs in response to COVID-19 spread are also not static, and thus the results of surveys can change over time [46]. For example, the adaptive type of response to a pandemic, in the form of a stress level reduction, observed in the initial stages of a pandemic, can be associated with an increase in awareness of a new infection [46], while the economic changes or the uncontrolled infection spread can cause an increase in stress levels. As a result, the prevalence of psychological stress among HCWs may increase after the initial period of an outbreak [8].

The differences in regulation and restriction measures among countries could strongly affect both the practice and mental health of HCWs. Thus, in Italy, all respondents reported practice closure or strong activity reduction, and the majority of them (89.6%) reported concerns about their professional future and the hope for economic measures to help dental practitioners [23]. A vast decrease in the number of treated patients was also observed in other countries as well [13,21].

In contrast to some other studies, no differences between females and males on the DASS-21, PSS-SR, and IES-R scores were revealed. This finding is similar to the results of cross-sectional surveys among dentists from China, India, Israel, Italy, and the U.K. [19], as well as U.K. frontline HCWs, where no gender differences were revealed [3]. By contrast, several other studies have revealed that female dentists showed significantly higher levels of self-reported anxiety [13], depression [12], and stress [21], confirming that women are at higher risk of depressive symptoms than men [47]. Therefore, our findings should be treated with caution, because the majority of participants identified as women (78.9%) and it may be that there was insufficient power to detect differences.

According to the conducted analyses, the highest DASS-21 depression, anxiety, and stress scores, as well as PSS-SR arousal scores, and IES-R avoidance and total scores, were revealed in HCWs aged 51–64. In contrast to our findings, in India [21] and Italy [12], age was negatively correlated with depressive symptoms. The probable explanation could lie in the fact that persons over 65 years were not permitted to work during the pandemic, and thus were not included in our study. Moreover, HCWs aged 51–64 (the pre-retirement age in Russia) are the most vulnerable to the economic situation [48] and therefore economic anxiety—levels during the pandemic are essentially equal to the health anxiety [49], and could affect their stress and depression scores. This explanation is in line with the findings of U Consolo et al. (2020), who revealed that the majority of dental practitioners were quite concerned about their professional future, due to the uncertainty about the end of the emergency [23].

According to the results (Table 2), 7–17.2% of dental HCWs had clinical symptoms of PTSD (using established cut-offs for the IES-R, and the PSS-SR, respectively). The revealed prevalence of PTSD symptoms is similar to that of comparable studies [32,33,34].

HCWs working in patient-facing roles had significantly higher PSS-SR re-experiencing scores, PSS-SR total scores, IES-R intrusion, IES-R avoidance scores, and IES-R total scores than their colleagues in non-patient-facing roles. Moreover, according to the multiple regression analysis (Table 3), work in a patient-facing role was a strong predictor for PTSD development. The obtained results are similar to those reported by other researchers, who identified differences in the depression scores of face-to-patient HCWs vs HCWs who were not seeing any patients [28] and established that patient-facing roles were the predictors of burnout [29]. These findings can be explained by the HCWs’ awareness of the higher chances for patient-facing specialists to become infected [4,50,51]. According to the previous surveys, the majority of practitioners feared infection, and that fear of contracting COVID-19 from a patient was strongly associated with elevated psychological distress [23,24].

Notably, an examination of the levels of PTSD, anxiety, and depression symptoms among the study groups have revealed that dentists (who hold MD degrees), in contrast to other dental HCWs, had significantly higher PSS-SR re-experiencing, arousal, intrusions, and total scores, while dental assistants (persons without an MD degree), in contrast to other participants, had significantly higher IES-R avoidance scores. This is somewhat interesting, because nurses (which is the closest analog to dental assistant in general medicine) typically reported higher levels of symptoms and distress than doctors [3,6,8,52], with a few studies reporting no difference [53,54] and only one study reporting higher rates in doctors [30].

Overall, our study has identified a vulnerable group susceptible to psychological distress. However, mental health problems have also been found to be associated with increased risk for cardiovascular diseases, diabetes, and even premature mortality [55], and thus future studies, as well as special clinical and policy strategies, are needed.

## 5. Conclusions

This study highlights that dental HCWs in Russia have high levels of psychological distress and PTSD symptoms during the COVID-19 pandemic outbreak. HCWs working directly with patients have significantly higher levels of PTSD symptoms and the risk of PTSD development compared to those working indirectly, whereas older HCWs have significantly higher levels of both psychological distress and PTSD symptoms compared to younger HCWs. Furthermore, dentists with MD degrees have significantly higher levels of PTSD symptoms compared to other dental HCWs, whereas dental assistants have significantly higher levels of the specific PTSD symptom avoidance, compared to other dental HCWs. Psychological support for vulnerable HCWs, including psychological prevention and early intervention, may be beneficial.

### Limitations of the Study

Due to the scale of the COVID-19 pandemic, this study has several limitations. Firstly, the study was subject to selection bias and sampling error, because all data were obtained from HCWs cohorts admitted in only three dental clinics in Ekaterinburg, Russia. The sample of participants was not representative, and therefore the study was more of a pilot in nature. Selection bias and response bias may have resulted in an overestimation or underestimation of psychological distress. The revealed effects may be different among HCWs in geographically diverse populations. Secondly, this study does not allow comparison to pre-pandemic baseline data. However, previous research suggests that HCWs experience higher rates of anxiety and depression when compared with the general population. Moreover, because of the Russian regulation rules, persons over 65 years of age were not permitted to work during the pandemic and this factor could influence the results of the survey. Therefore, large-scale prospective cohort studies will be required in ethnically and geographically diverse cohorts to better understand the prevalence and risk factors for psychological distress among dentist specialists.

## Figures and Tables

**Table 1:** Participant demographic characteristics.

Variable	Frequency, n (%)
Sex	
Male	27 (21.1)
Female	101 (78.9)
Age	
21–35 years	54 (42.2)
36–50 years	54 (42.2)
51–64 years	20 (15.6)
Working position	
Direct	80 (62.5)
Indirect	48 (37.5)
Occupation	
Dentists	43 (33.6)
Dental Assistants	37 (28.9)
Dental auxiliaries	48 (37.5)

**Table 2:** Prevalence of psychological distress and PTSD symptoms in healthcare workers.

Category	Total	Sex		Age			Working Position		Occupation		
		M	F	21–35 Years	36–50 Years	51–64 Years	Dir.	Indir.	Dent.	Dent. ass.	Dent. auxil.
Prevalence, %											
DASS-21 dep.											
normal	79.69	81.48	79.21	85.19	87.04	45.00	80.00	79.17	79.07	81.08	79.17
mild	11.72	11.11	11.88	9.26	9.26	25.00	10.00	14.58	9.30	10.81	14.58
moderate	6.25	3.70	6.93	3.70	0	30.00	8.75	2.08	9.30	8.11	2.08
severe	1.56	3.70	0.99	1.85	1.85	0	1.25	2.08	2.33	0	2.08
extreme severe	0.78	0	0.99	0	1.85	0	0	2.08	0	0	2.08
DASS-21 anx.											
normal	75.78	66.67	78.22	81.48	74.07	65.00	78.75	70.83	79.07	78.38	70.83
mild	14.84	22.22	12.87	11.11	14.81	25.00	12.50	18.75	9.30	16.22	18.75
moderate	3.91	7.41	2.97	1.85	5.56	5.00	3.75	4.17	4.65	2.70	4.17
severe	3.12	3.70	2.97	5.56	1.85	0	2.50	4.17	2.33	2.70	4.17
extreme severe	2.34	0	2.97	0	3.70	5.00	2.50	2.08	4.65	0	2.08
DASS-21 str.											
normal	75.78	70.37	77.23	81.48	79.63	50.00	76.25	75.00	69.77	83.78	75.00
mild	11.72	14.81	10.89	3.70	16.67	20.00	10.00	14.58	16.28	2.70	14.58
moderate	7.81	11.11	6.93	14.81	0	10.00	7.50	8.33	4.65	10.81	8.33
severe	3.91	3.70	3.96	0	1.85	20.00	6.25	0	9.30	2.70	0
extreme severe	0.78	0	0.99	0	1.85	0	0	2.08	0	0	2.08
PSS-SR tot.											
mild	70.31	70.37	70.30	74.07	74.07	50.00	65.00	79.17	58.14	72.97	79.17
moderate	22.66	22.22	22.77	18.52	25.93	25.00	25.00	18.75	30.23	18.92	18.75
severe	7.03	7.41	6.93	7.41	0	25	10.00	2.08	11.63	8.11	2.08
IES-R tot.											
normal	92.97	96.30	92.08	100	90.74	80.00	92.50	93.75	90.70	94.59	93.75
mild	3.91	0	4.95	0	5.56	10.00	2.50	6.25	4.65	0	6.25
moderate	1.56	3.70	0.99	0	3.70	0	2.50	0	2.33	2.70	0
severe	1.56	0	1.98	0	0	10.00	2.5	0	2.33	2.70	0

**Table 3:** Descriptive statistics for DASS-21, PSS-SR, and IES-R scores.

Category	Total	Sex		Age			Working Position		Occupation		
		M	F	21–35 Years	36–50 Years	51–64 Years	Dir.	Indir.	Dent.	Dent. ass.	Dental auxil.
Mean (SD) DASS-21, PSS-SR, and IES-R Scores											
DASS-21 dep.	2.63	2.96	2.55	2.11	2.41	4.65	2.56	2.75	2.77	2.32	2.75
SD	2.98	2.86	3.02	2.70	2.81	3.44	2.83	3.25	3.02	2.61	3.25
DASS-21 anx.	2.23	2.41	2.19	1.59	2.52	3.20	2.09	2.48	2.33	1.81	2.48
SD	2.66	2.36	2.74	2.25	2.85	2.84	2.68	2.63	3.10	2.11	2.63
DASS-21 stress	4.48	4.7	4.40	3.76	4.26	7.00	4.61	4.25	5.33	3.78	4.25
SD	4.06	4.30	4.01	3.78	3.78	4.69	4.05	4.11	4.32	3.58	4.11
DASS-21 total	9.34	10.15	9.13	7.46	9.19	14.8	9.26	9.48	10.4	7.92	9.48
SD	8.88	8.21	9.08	8.14	8.72	9.38	8.71	9.25	9.45	7.67	9.25
PSS-SR re-exp.	1.7	1.33	1.88	1.52	1.59	2.90	2.13	1.17	2.23	2.00	1.17
SD	2.14	1.39	2.29	2.01	1.90	2.77	2.38	1.51	2.39	2.39	1.51
PSS-SR avoid.	2.6	2.41	2.75	2.57	2.19	4.30	3.01	2.13	3.16	2.84	2.13
SD	3.11	3.21	3.10	3.40	2.08	4.13	3.32	2.69	3.36	3.30	2.69
PSS-SR arousal	2.7	2.89	2.73	2.26	2.69	4.35	3.08	2.25	3.56	2.51	2.25
SD	2.53	2.75	2.48	2.30	2.18	3.38	2.70	2.14	2.88	2.40	2.14
PSS-SR total	7.2	6.63	7.37	6.35	6.46	11.5	8.21	5.54	8.95	7.35	5.54
SD	6.88	6.49	2.48	6.61	5.35	9.53	7.38	5.64	7.72	7.00	5.64
IES-R intrus.	2.4	1.89	2.55	1.57	2.63	4.10	2.85	1.69	3.09	2.57	1.69
SD	3.41	2.71	3.57	2.63	3.54	5.04	3.59	2.98	3.87	3.25	2.98
IES-R avoid.	3.1	2.44	3.29	2.35	2.94	5.60	3.68	2.17	3.60	3.76	2.17
SD	3.92	3.51	4.02	3.05	3.91	5.06	4.18	3.25	4.34	4.05	3.25
IES-R hyperar.	2.0	2.37	1.99	1.59	1.91	3.80	2.29	1.71	2.49	2.05	1.71
SD	2.74	2.47	2.82	2.13	2.51	4.02	3.04	2.14	3.27	2.78	2.14
IES-R total	7.5	6.70	7.83	5.52	7.48	13.5	8.81	5.56	9.19	8.38	5.56
SD	9.32	8.02	9.65	6.20	9.41	13.28	10.04	7.63	10.81	9.19	7.63

DASS-21—Depression Anxiety and Stress Scale-21 [36]; IES-R--Event Scale-Revised [39]; PSS-SR—PTSD Symptom Scale-Self-Report [42].

**Table 4:** The risk factors for PTSD symptoms among healthcare workers.

Model	Β	95%CI	SE	β	t	*p*
	Model 1. Predictors of PTSD Symptoms (PSS-SR)					
Sex	0.37	−1.64 to 2.39	1.02	0.05	0.37	>0.05
Working position	2.54	0.82 to 4.26	0.87	0.37	1.31	<0.01
Age	0.01	−0.07 to 0.08	0.04	0.01	0.13	>0.05
Depression	0.49	−0.03 to 1.00	0.26	0.21	1.87	>0.05
Anxiety	0.39	−0.07 to 0.85	0.23	0.15	1.68	>0.05
Stress	0.76	0.42 to 1.10	0.17	0.45	4.34	<0.001
	Model 2. Predictors of PTSD Symptoms (IES-R)					
Sex	0.66	−2.46 to 3.77	1.57	0.07	0.31	>0.05
Working position	2.87	0.22 to 5.53	1.34	0.31	1.59	<0.05
Age	0.06	−0.07 to 0.18	0.06	0.06	0.86	>0.05
Depression	0.47	−0.32 to 1.27	0.40	0.15	1.10	>0.05
Anxiety	0.40	−0.31 to 1.11	0.36	0.11	1.12	>0.05
Stress	1.00	0.48 to 1.52	0.27	0.44	3.83	<0.001

## Data Availability

The datasets analyzed during the current study are available from the corresponding author on reasonable request as they contain information on the gender, age, work experience, and places of work of the respondents.
